# Editorial: Shankopathies: Shank Protein Deficiency-Induced Synaptic Diseases

**DOI:** 10.3389/fnmol.2020.00011

**Published:** 2020-02-07

**Authors:** Elodie Ey, Thomas Bourgeron, Tobias M. Boeckers, Eunjoon Kim, Kihoon Han

**Affiliations:** ^1^Human Genetics and Cognitive Functions, Institut Pasteur, UMR 3571 CNRS, Université de Paris, Paris, France; ^2^Institute for Anatomy and Cell Biology, Ulm University, Ulm, Germany; ^3^Center for Synaptic Brain Dysfunctions, Institute for Basic Science, Daejeon, South Korea; ^4^Department of Biological Sciences, Korea Advanced Institute of Science and Technology, Daejeon, South Korea; ^5^Department of Neuroscience, and Biomedical Sciences, Korea University College of Medicine, Seoul, South Korea

**Keywords:** Shank, Shankopathies, neuropsychiatric disorders, modulating factors, brain regions

SHANK (also known as ProSAP) proteins are postsynaptic core scaffolds involved in excitatory synapse development, function, and plasticity. The three members of the SHANK family (SHANK1, SHANK2, and SHANK3) differ in their temporal and regional expression patterns in the central nervous system and non-neuronal tissue during development. Mutations in the cognate genes are associated with various neuropsychiatric conditions, such as autism spectrum disorders, Phelan-McDermid syndrome, intellectual disability, schizophrenia, and bipolar disorder. Exactly how defects in SHANK proteins contribute to these conditions is currently under active investigation.

The present Research Topic provides an overview of current knowledge on the involvement of SHANK genes in neuropsychiatric disorders. Shankopathies are explored at different scales: genes, proteins, cells, synapses, neural circuits, behaviors, and environment. For example, Eltokhi et al. observed that the large phenotypic diversity of patients carrying *SHANK2* mutations is reflected in the phenotypic diversity displayed by the various *Shank2* mouse models. Hassani Nia and Kreienkamp reviewed the effects of *Shank3* mutations on the gene, protein, and synaptic signaling. Both reviews provide a comprehensive state-of-the-art overview on *SHANK2* and *SHANK3* different scales. An example of how mouse models can closely mimic human Shankopathies was provided by the comprehensive characterization of the *Shank3*^Q321R^ knock-in model conducted by Yoo et al..

The other contributions address two major questions for Shankopathies: (i) Are there specific modulating factors for Shankopathies and (ii) Which brain regions are specifically affected by Shankopathies?

Concerning the first question, three putative modulating factors were examined; namely, pharmacological intervention (pharmacological modulation of phenotypes), sexual hormones, and zinc. Sungur et al. explored whether *Shank1* mutant mice and control mice were equally sensitive to amphetamine and methylenedioxymethamphetamine. In *Shank2* mutant mice, Ey et al. demonstrated that the abnormal social behavior of *Shank2* knockout mice was associated with a deficit in social motivation, but with an intact social recognition phenotype. They also highlighted that methylphenidate is ineffective in restoring typical activity levels in hyperactive *Shank2* knockout mice. By contrast, Berkel et al. examined the role of an inherent modulating factor; namely, sexual hormones, on *Shank1, Shank2*, and *Shank3* gene transcription and protein levels. The role of sexual hormones as transcriptional fine-tuners, especially shortly before or after birth, might explain sex-related differences in patients and animal models. Finally, zinc was identified as a potentially major modulating factor in Shankopathies, given its role in recruiting Shank2 and Shank3 at synapses, as well as having a key role in the gastro-intestinal tract, as reviewed by Hagmeyer et al.. At the molecular level, the mode of regulation of AMPA receptor (AMPAR) subunit composition by *Shank2, Shank3* and zinc was explored by Ha et al.. Zinc regulated the switch during development of AMPARs lacking GluA2 to AMPARs containing GluA2, while Shank2 and Shank3 also regulated this process. At the scale of the organism, Fourie et al. tested the effect of long-term zinc supplementation in alimentation of the *Shank3*^Δ*ex*13^^−16^ mouse model. Importantly, some aspects of synaptic transmission and some behavioral traits, such as self-grooming, were rescued. Taken together, the studies on zinc presented in this Research Topic present evidence that zinc is a promising potential new therapeutic agent for Shankopathies.

Regarding the second question on brain regions specifically affected by Shankopathies, different approaches were used to investigate the effect of *Shank* mutations at the different scales of observation. Yoo et al. highlighted that a conditional *Shank3* knockout (targeting exons 14–16, encoding the PDZ domain) in GABAergic neurons (enriched in the striatum) induces a strong reduction of excitatory synaptic inputs onto dorsal striatal neurons as observed in global *Shank3*^Δ*ex*14^^−16^ mutant mice. However, the conditional knockout leads to milder social deficits and stereotyped behaviors than global *Shank3*^Δ*ex*14^^−16^ mutant mice. Using magnetic resonance imaging (MRI), Schoen et al. examined mouse brain anatomy during development showing that total brain volume, cerebellar volume, and cortical thickness were unaffected in homozygous and heterozygous *Shank3* mutant mice and the prenatal zinc deficiency model compared to wild-type controls. By contrast, the striatal volume and the globus pallidus volume were increased in both models, while the changes in the thalamus were reduced in the *Shank3* mouse model and increased in the prenatal zinc deficiency mouse model. Transcriptomic analyses allowed (Jin et al.) to identify brain region-specific modulation of gene expression in different *Shank3* models, across age classes and between brain regions (prefrontal cortex, striatum, and hippocampus). This analysis highlighted an important modulation of the expression of myelin-related and ribosome-related genes. At the protein level, Heise et al. found that *Shank2* and *Shank3* mutant mice displayed a reduced expression of receptors specific to excitatory synaptic transmission in the striatum and in the thalamus (for *Shank2* also in the cortex and cerebellum). These models were compared to another mouse model of neurodevelopmental disorders, the *Cntn4* mutant that displays increased surface expression of glutamatergic receptors (GluA1 and GluA2) in the striatum or downregulation of GABAergic receptors (GABAA) in several brain regions. Finally, the involvement of the reward system was examined directly and indirectly. In the direct approach, Bariselli et al. knocked down *Shank3* in the ventral tegmental area in mice and observed a dysfunction in social-seeking behavior. The indirect approach concerned the characterization of a *Shank2* model in another species, namely the *Shank2*^Δ*ex*31^ mutant rat, characterized by Modi et al.. *Shank2*^Δ*ex*31^ mutant rats displayed an atypically high motivation to collect food reward, in parallel with social deficits, impaired learning, increased activity, and repetitive circling, combined with increased striatal activity and decreased hippocampal function.

In summary, this Research Topic provided a multi-scale overview of the current state of knowledge on Shankopathies. The research studies contribute to the understanding of the spectrum of disorders related to *SHANK* mutations, as well as providing prospects for new potential therapeutic strategies, targeting specific brain regions or modulating factors.

## Author Contributions

All authors listed have made a substantial, direct and intellectual contribution to the work, and approved it for publication.

### Conflict of Interest

The authors declare that the research was conducted in the absence of any commercial or financial relationships that could be construed as a potential conflict of interest.

